# H1Innovative approaches to combat anti‐cancer drug resistance: Targeting lncRNA and autophagy

**DOI:** 10.1002/ctm2.1445

**Published:** 2023-10-14

**Authors:** Chenming Zhong, Zijun Xie, Shiwei Duan

**Affiliations:** ^1^ Key Laboratory of Novel Targets and Drug Study for Neural Repair of Zhejiang Province, School of Medicine Hangzhou City University Hangzhou Zhejiang P. R. China; ^2^ Medical Genetics Center School of Medicine Ningbo University Ningbo Zhejiang P. R. China

**Keywords:** apoptosis, autophagy, cancer, drug resistance, lncRNA

## Abstract

**Background:**

To date, standardizing clinical predictive biomarkers for assessing the response to immunotherapy remains challenging due to variations in personal genetic signatures, tumour microenvironment complexities and epigenetic onco‐mechanisms.

**Main Body:**

Early monitoring of key non‐coding RNA (ncRNA) biomarkers may help in predicting the clinical efficacy of cancer immunotherapy and come up with standard predictive ncRNA biomarkers. For instance, reduced miR‐125b‐5p level in the plasma of non‐small cell lung cancer patients treated with anti‐PD‐1 predicts a positive outcome. The level of miR‐153 in the plasma of colorectal cancer patients treated with chimeric antigen receptor T lymphocyte (CAR‐T) cell therapy may indicate the activation of T‐cell killing activity. miR‐148a‐3p and miR‐375 levels may forecast favourable responses to CAR‐T‐cell therapy in B‐cell acute lymphoblastic leukaemia. In cancer patients treated with the GPC3 peptide vaccine, serum levels of miR‐1228‐5p, miR‐193a‐5p and miR‐375‐3p were reported as predictive biomarkers of good response and improved overall survival. Therefore, there is a critical need for further studies to elaborate on the key ncRNA biomarkers that have the potential to predict early clinical responses to immunotherapy.

**Conclusions:**

This review summarises important predictive ncRNA biomarkers that were reported in cancer patients treated with different immunotherapeutic modalities including monoclonal antibodies, small molecule inhibitors, cancer vaccines and CAR‐T cells. In addition, a concise discussion on forthcoming perspectives is provided, outlining technical approaches for the optimal utilisation of immune‐modulatory ncRNA biomarkers as predictive tools and therapeutic targets.

## Graphical abstract

Over 30 lncRNAs impact autophagy in 16 tumour cell types, contributing to drug resistance.

Thirteen anti‐cancer drugs are influenced by lncRNAs and autophagy in drug resistance.

Multiple lncRNAs concurrently regulate cellular autophagy, impacting resistance to a single drug.

The lncRNA‐autophagy axis presents novel avenues for enhancing chemotherapy's efficacy.

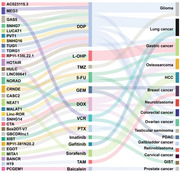

## 1 INTRODUCTION

In 1963, Christian de Duve first introduced the term ‘autophagy’, leading to increased attention and research on this mechanism.^1^ ^,2^ In 2016, Yoshinori Ohsumi received the Nobel Prize in Physiology or Medicine for his ground‐breaking research that elucidated the mechanism of autophagy, underscoring its critical relevance in the context of human health and the treatment of diseases.^1^ Autophagy is a cellular process wherein components like proteins and organelles are conveyed to lysosomes to undergo degradation. It includes three main types: macroautophagy, microautophagy and chaperone‐mediated autophagy (CMA).^3^, ^4^ Macroautophagy, the most extensively examined form of autophagy, entails the creation of double‐membrane autophagic vesicles designed to encapsulate and degrade organelles and proteins within lysosomes.^5^

Recent research has shown that abnormal regulation of autophagy is involved in the development of various tumours. Autophagy plays a dual role, inhibiting tumour progression in the early stages and promoting it in the later stages.^5^, ^6^ Based on this role, autophagy‐targeting drugs have been developed and used in anti‐tumour therapy, most of which inhibit autophagy. Overcoming drug resistance in tumour chemotherapy is a major challenge. Acquired drug resistance often occurs when drugs are initially effective but their efficacy decreases over time.^7^ The abnormal autophagy process has also been found to play a role in the resistance to existing anti‐cancer drugs.^8^

Non‐coding RNAs, including lncRNAs, microRNAs (miRNAs) and circular RNAs (circRNAs), constitute a diverse group. These non‐coding RNAs coexist within intricate and intertwined regulatory networks, often exerting either similar or opposing effects on various biological processes throughout tumour development and progression.^9^ LncRNA has become a research hotspot in recent years. Numerous lncRNAs have been discovered to function as either oncogenes or tumour suppressor genes during the development of cancer, and their anomalous expression and genetic mutations are intricately linked to the processes of tumourigenesis, metastasis and tumour progression.^10^ LncRNAs are known to participate in a myriad of cellular functions, encompassing autophagy, proliferation, migration, invasion, epithelial to mesenchymal transition (EMT) and apoptosis.^9^ Moreover, recent studies have illuminated their significant roles in various cell death mechanisms within tumours including pyroptosis, ferroptosis and copper death.^11^, ^12^, ^13^ LncRNAs exert their influence on tumour progression through various mechanisms. These include interactions with RNA‐binding proteins,^10^ competitive inhibition of miRNAs,^14^ aberrant epigenetic modifications,^15^, ^16^ and regulation of cancer stem cells (CSCs).^17^ By utilizing these mechanisms, lncRNAs can target crucial genes involved in different stages of autophagy, modulating their expression and subsequently promoting or inhibiting autophagy. Consequently, these processes have a direct impact on tumour progression.^18^ Abnormal expression of many lncRNAs has been found to be related to resistance to anti‐tumour drugs. For example, MIR4435‐2HG promotes cisplatin (DDP) resistance in non‐small cell lung cancer (NSCLC) and colon cancer .^19^ LINC00665 enhances resistance to gefitinib and DDP in NSCLC and gemcitabine (GEM) in cholangiocarcinoma.^20^

This review explores whether the generation of anti‐tumour drug resistance is mediated by abnormal lncRNA expression and autophagy and whether there is a direct connection and regulatory relationship between the two. It concludes that lncRNAs can regulate autophagy through multiple mechanisms and participate in the resistance of at least 13 anti‐cancer drugs. The cross‐talk relationship between autophagy and apoptosis in drug resistance is also explored. The aim of this research is to focus on the impact of lncRNAs and autophagy on drug resistance and sensitivity in tumour chemotherapy, contributing to the improvement of existing chemotherapy regimens and the development of more targeted and effective therapeutic strategies.

## 2 AUTOPHAGY AND CANCER

### 2.1 An overview and basic molecular mechanism of autophagy

**FIGURE 1:**
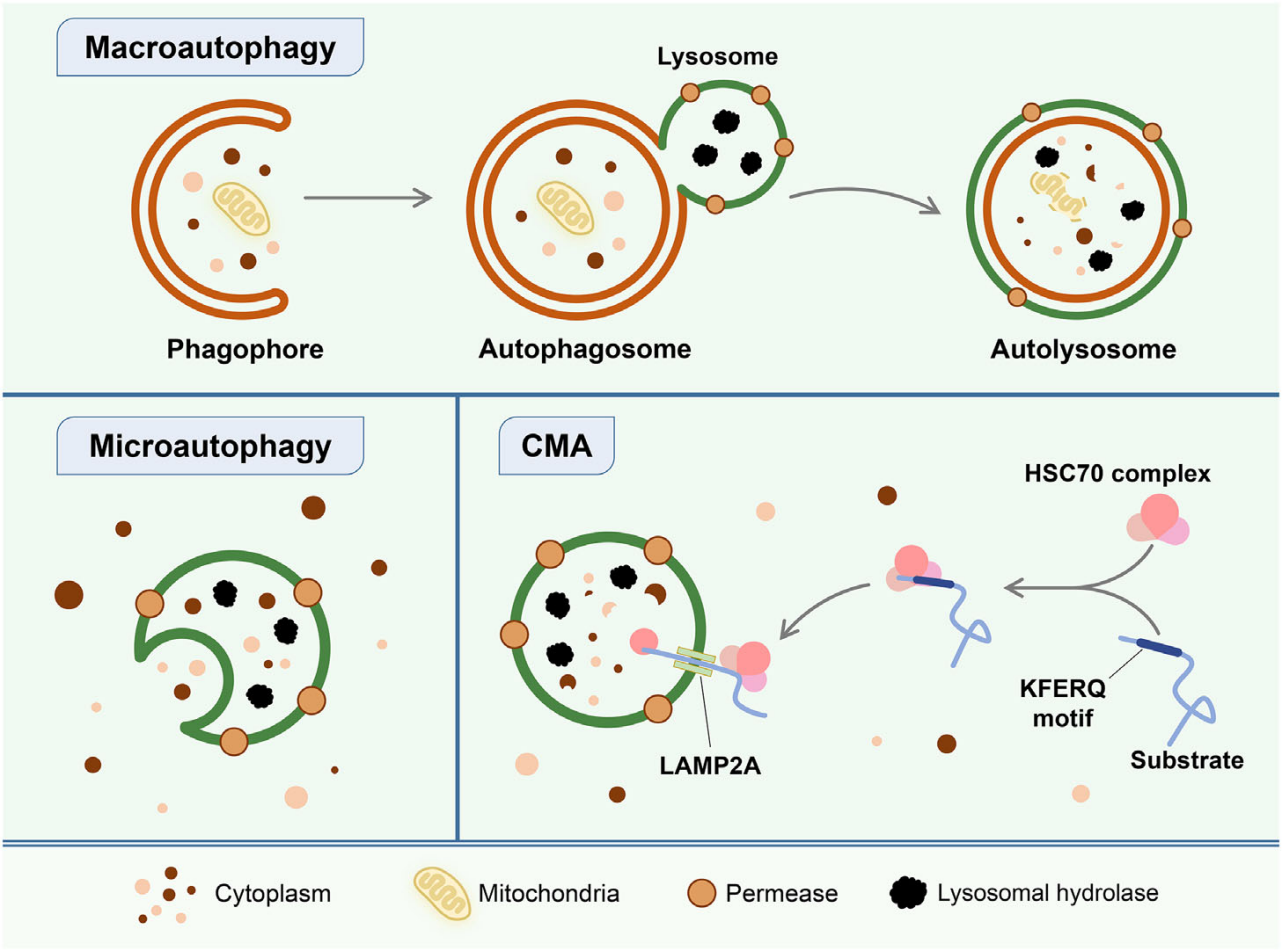
Three types of autophagy. Autophagy can be classified into three main types: macroautophagy, microautophagy and chaperone‐mediated autophagy (CMA). Macroautophagy involves the formation of double‐membrane vesicles called autophagosomes in the cytoplasm, which sequester and transport proteins and organelles to lysosomes for degradation. In contrast, microautophagy involves the direct uptake of cytoplasmic contents by lysosomes through membrane invagination or deformation. CMA targets specific substrates containing a KFERQ‐like motif. These substrates bind to the cytoplasmic chaperone HSC70 and are transported into the lysosomal cavity via lysosomal‐associated membrane protein 2A (LAMP2A).

**FIGURE 2:**
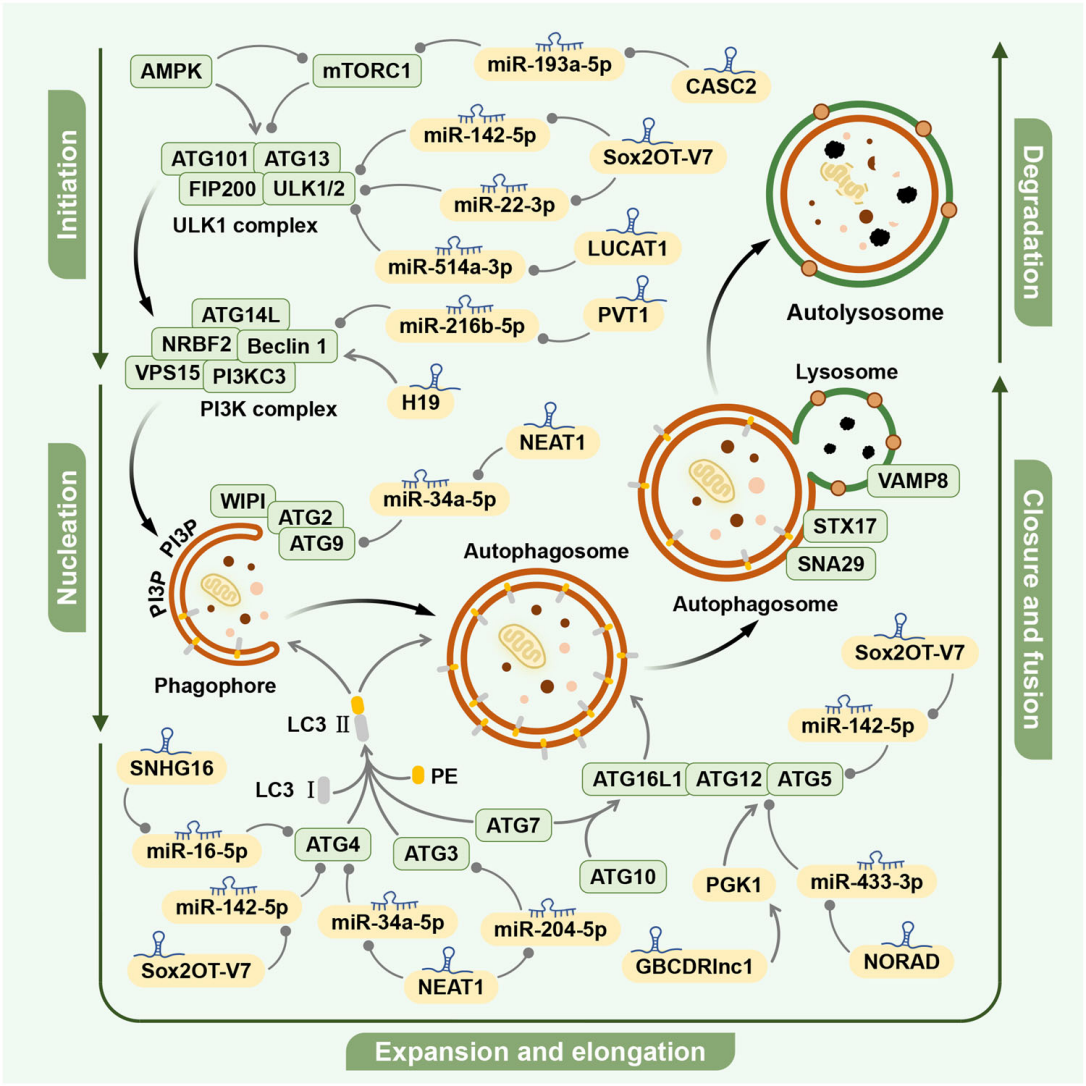
The five stages of macroautophagy and the role of lncRNAs in regulating autophagy. Macroautophagy is a complex process that can be divided into five stages: initiation, nucleation, expansion and elongation, closure and fusion and degradation. Many lncRNAs have been shown to regulate autophagy by targeting key regulators in the autophagy process. This can mediate tumor drug resistance.

The term ‘autophagy’ comes from the Greek roots ‘auto’ and ‘phagy’, meaning ‘self’ and ‘eating’, respectively.^21^ Autophagy is a highly conserved cellular catabolic pathway that includes three main types: macroautophagy, microautophagy and CMA.^4^ Macroautophagy involves the formation of double‐membrane vesicles called autophagosomes that capture cytoplasmic organelles and macromolecular complexes.^1^ Microautophagy involves the direct uptake of cytoplasmic contents by lysosomes through membrane invagination or deformation. CMA is highly selective and involves the binding of substrates with a specific KFERQ‐like motif to the cytoplasmic partner HSC70 and transport into lysosomes mediated by lysosomal‐associated membrane protein 2A (LAMP2A).^4^, ^22^ All three types ultimately transport specific substances in the cytoplasm to lysosomes for degradation and recycling (Figure 1).^23^ In most cases, autophagy refers to macroautophagy, which is the most widely studied form.^1^ The autophagy process unfolds through a series of discrete phases, which include initiation, the formation of autophagosome precursors, the enlargement and elongation of autophagosome membranes, the sealing and merging with lysosomes and the degradation of enclosed contents.^1^ Each of these stages is meticulously controlled by a designated ensemble of autophagy‐related (ATG) genes and accompanying proteins (Figure 2).^24^

In the initial stage of autophagy, the activation of the Unc‐51‐like kinase (ULK) complex is crucial. This complex includes ULK1, ULK2, ATG13, ATG101 and focal adhesion kinase‐interacting protein 200 kDa (FIP200). The ULK complex is generally inactive but becomes active when cells are under certain stress conditions such as starvation, nutrient deprivation, hypoxia and DNA damage. This activation also occurs when the mammalian target of rapamycin complex 1 is inhibited or AMP‐activated protein kinase (AMPK) is activated.^25^, ^26^

Then, the ULK1 complex reactivates class III phosphatidylinositol 3‐kinase (PI3K) complexes including PI3KC3 (Vps34 in yeast), Beclin 1, VPS15 (PIK3R4), NRBF2 (Atg38 in yeast) and ATG14L. This triggers vesicle nucleation.^1^, ^3^, ^27^ PI3KC3 generates phosphatidylinositol‐3‐phosphate (PI3P) and stabilizes the ULK1 complex. At the site of PI3P generation, WIPI protein and its binding partner ATG2A or ATG2B play a role in the early stage of membrane elongation.^3^, ^28^ ATG9 is the only integral membrane protein in the core autophagy mechanism and cooperates with ATG2 to transport and supply lipids. However, its specific mechanism still requires further study.^29^

The expansion and elongation of the autophagosome membrane are mediated by two ATG7‐dependent ubiquitin‐like coupling systems. These systems include the formation of the ATG12‐ATG5‐ATG16L1 complex and the coupling of phosphatidylethanolamine (PE) to LC3.^24^ LC3 is processed at its C‐terminus by ATG4B to become LC3‐I. Then, through an ubiquitination‐like enzymatic reaction mediated by the E1‐like enzyme ATG7 and the E2‐like enzyme ATG3, LC3‐I combines with PE to become LC3‐II. LC3‐II can be used as a measure of autophagic flux.^24^

The fusion of autophagosomes and lysosomes is mediated by STX17 and SNAP29 on autophagosomes, vesicle‐associated membrane protein 8 (VAMP8) on lysosomes and the homotypic fusion and protein sorting (HOPS) complex.^26^ Ultimately, acid hydrolases housed within lysosomes break down the autophagic cargo, releasing the reclaimed nutrients back into the cytoplasm for the cell's subsequent utilization.^26^

A multitude of studies have convincingly showcased the regulatory influence of lncRNAs on autophagy within tumours, thereby influencing the development of drug resistance. Notably, a significant body of research has focused on the competing endogenous RNAs (ceRNA) mechanism, wherein lncRNAs target and inhibit miRNAs to modulate the expression of downstream autophagy‐related molecules.^16^, ^30^, ^31^, ^32^, ^33^, ^34^, ^35^, ^36^ Moreover, lncRNAs can directly impact downstream proteins, as exemplified by the upregulation of phosphoglycerate kinase 1 (PGK1) facilitated by the high expression of GBCDRlnc1.^37^, ^38^ Furthermore, H19 has been shown to impede the methylation of the critical molecule Beclin 1, leading to its enhanced expression.^15^ These findings underscore the diverse mechanisms through which lncRNAs exert their influence on autophagy‐related pathways and contribute to drug resistance.

### 2.2 The dual roles of autophagy in tumour progression

The association between autophagy and cancer was initially established in 1999 with the discovery that Beclin 1, encoded by the BECN1 gene, facilitates autophagy in human MCF‐7 breast cancer cells and inhibits tumour cell proliferation.^39^ Subsequently, Qu et al. provided further evidence by demonstrating that mice with heterozygous deletion of the BECN1 gene exhibited a substantially higher incidence of spontaneous tumours compared to wild‐type mice.^40^ These seminal findings underscore the critical role swof Beclin 1‐mediated autowphagy in cancer development and highlight the significance of understanding this molecular pathway for potential therapeutic interventions. However, subsequent studies have found that autophagy has seemingly contradictory dual effects on the survival or death of tumour cells at different stages of tumour development. This is related to multiple factors such as the type and stage of cancer, genetic background, tumour microenvironment and immunity.^6^, ^41^, ^42^ In the early stages of cancer progression, autophagy removes oncogenic proteins, maintains genome stability, induces stress‐related responses and immune response mechanisms to prevent tumour proliferation, invasion and metastasis. As cancer progresses to advanced stages, autophagy can provide nutrients and energy for tumour cells and maintain functional mitochondria, reduce DNA damage and enhance cancer cells' response to stress. This plays a tumour‐promoting role.^6^, ^41^, ^42^, ^43^

There is a lot of research focused on developing more specific and effective autophagy‐targeted drugs to treat cancer. However, the complex dual role of autophagy in tumours has increased the difficulty of this therapeutic strategy. Most existing research focuses on inhibiting autophagy.^43^, ^44^ Since autophagy is a multi‐step process, many links in it can become potential drug targets.^1^ Currently, chloroquine (CQ) and hydroxychloroquine (HCQ) are the only clinically available antineoplastic agents that inhibit autophagy by de‐acidifying lysosomes and preventing fusion of autophagosomes with lysosomes. There is also evidence that drugs targeting autophagy regulators in other stages of autophagy, such as ULK1/2, VPS34 and ATG4B, can have anti‐tumour effects. These drugs are currently in the pre‐clinical stage and require further experimental research and clinical verification.^1^, ^44^ Furthermore, there is growing evidence suggesting that the stimulation of autophagy could potentially serve as a viable approach for cancer treatment. For instance, in the case of glioma, the combined administration of tricyclic anti‐depressant imipramine and purinergic receptor inhibitor ticlopidine has been shown to enhance autophagy and induce autophagy‐dependent cell death.^45^ Furthermore, recent research highlights the potential of nanomaterials to serve as carriers in conjunction with autophagy regulators, thereby enhancing their bioavailability in cancer treatment. Notably, certain nanoparticles have demonstrated the capacity to actively modulate the autophagy process within tumour cells through diverse mechanisms.^46^

The problem of drug resistance in cancer chemotherapy has attracted increasing attention. Abnormal regulation of tumour cell autophagy has been found to be related to the resistance of various anti‐cancer drugs. Autophagy also has a dual role in the development of chemotherapeutic drug resistance, either promoting or suppressing resistance.^47^ Autophagy may be an effective cancer escape mechanism in most tumours. Drug treatment induces enhanced autophagy, which mediates chemoresistance.^1^, ^8^ In a study conducted by Zhu et al., it was demonstrated that doxorubicin (DOX) treatment in the osteosarcoma cell line U2OS led to a dose‐dependent increase in the protein levels of LC3II and Beclin 1. Notably, when the autophagy inhibitor 3‐methyladenine (3‐MA) was administered in combination with DOX, a significant enhancement of the inhibitory effect on U2OS cell viability was observed.^31^ Similarly, in glioma, the administration of chemotherapeutic agents temozolomide (TMZ) and cisplatin (DDP) was found to induce the conversion of LC3‐I to LC3‐II, indicating an augmentation of autophagosome formation.^30^, ^48^, ^49^ These findings underscore the potential role of autophagy modulation in improving the efficacy of chemotherapy in different cancer types. Drug resistance mediated by autophagy is a complex phenomenon characterized by various factors, including the recycling of cytoplasmic components, genetic repair mechanisms, alterations in drug concentrations and metabolism, modifications in the expression or activity of crucial proteins, as well as shifts in apoptosis and survival signalling pathways.^47^ Certain types of tumour cells characterized by inherently elevated levels of autophagy might develop inherent resistance to chemotherapy and other targeted therapeutic approaches. ^47^ However, prolonged autophagy enhancement may also lead to cell death, suppressing drug resistance through autophagic cell death, independent of apoptosis and necrosis.^47^, ^50^

Autophagy plays a substantial role in conferring resistance to anti‐cancer drugs, and the combination of targeted autophagy with chemotherapy drugs has emerged as a promising research direction to mitigate drug resistance. Researchers are actively conducting clinical trials by combining autophagy inhibitors with chemotherapy drugs. For instance, in children with vemurafenib‐resistant brain tumours (a kinase inhibitor), the combination of CQ and vemurafenib has shown potential in overcoming drug resistance.^51^ However, considering the dual role of autophagy in tumour cells and its physiological role in maintaining homeostasis, it is crucial to understand the specific effects of autophagy in different types and stages of tumours. Precancerous lesions, primary tumours and metastatic tumours require cautious consideration regarding the timing and extent of autophagy modulation when using targeted drugs alone or in combination with chemotherapy drugs. Additionally, the current determination of the role of autophagy in cancer relies on detecting LC3 puncta and LC3‐II in tumour tissue, which only indicates the accumulation of autophagosomes without distinguishing autophagosome maturation arrest from enhanced induction of autophagy. This poses challenges for further studying autophagy in cancer, highlighting the need for new and more appropriate pharmacodynamic biomarkers.^1^, ^44^

## 3 LncRNA PARTICIPATES IN THE GENERATION OF ANTI‐CANCER DRUG RESISTANCE BY REGULATING AUTOPHAGY

LncRNAs are ncRNAs that are longer than 200 nucleotides and possess minimal to negligible protein‐coding potential. They are susceptible to various post‐transcriptional modifications, including 5′‐capping, 3′‐polyadenylation and splicing.^14^, ^52^, ^53^ Many lncRNAs have been found to function as oncogenes or tumour suppressor genes in various cancers. These molecules can exert control over gene expression in multiple ways, operating within the nucleus to influence epigenetic and transcriptional processes and also operating in the cytoplasm to impact post‐transcriptional and translational events. Consequently, lncRNAs exert their influence on several tumour‐related biological processes including proliferation, invasion, metastasis, apoptosis, autophagy and angiogenesis.^53^, ^54^ Concurrently, research has highlighted the connections between lncRNAs and diverse modes of cell death in tumour cells such as apoptosis, autophagy, pyroptosis, ferroptosis and copper death.^11^, ^12^, ^13^, ^54^

While many anti‐tumour drugs are effective in the early stages of treatment, they often eventually lead to acquired drug resistance. The abnormal expression of lncRNAs has been linked to the development of drug resistance.^7^, ^9^ LncRNAs play diverse roles in gene regulation. They can interact with RNA‐binding proteins, function as ceRNAs for miRNAs and participate in epigenetic modifications of DNA and RNA, thereby influencing the expression of associated genes. This can induce or inhibit tumour cell autophagy and mediate drug resistance.^10^, ^14^, ^47^ Furthermore, studies have elucidated that lysosomes have the capability to sequester hydrophobic weakly basic drugs, such as DOX, daunorubicin and sunitinib, thereby leading to multi‐drug resistance in chemotherapy.^55^ The fusion of autophagosomes and lysosomes represents a pivotal step in the autophagy process, affirming that targeting autophagy through lncRNA regulation could offer a viable approach to combat drug resistance.

In most cases, lncRNA and/or drug‐induced autophagy enhancement has been found to mediate drug resistance for 13 anti‐tumour drugs in at least 16 types of tumours. However, some researchers have also observed that autophagy was inhibited in DOX‐resistant neuroblastoma,^56^ paclitaxel (PTX)‐resistant breast cancer^57^ and baicalein‐resistant prostate cancer.^58^

**FIGURE 3:**
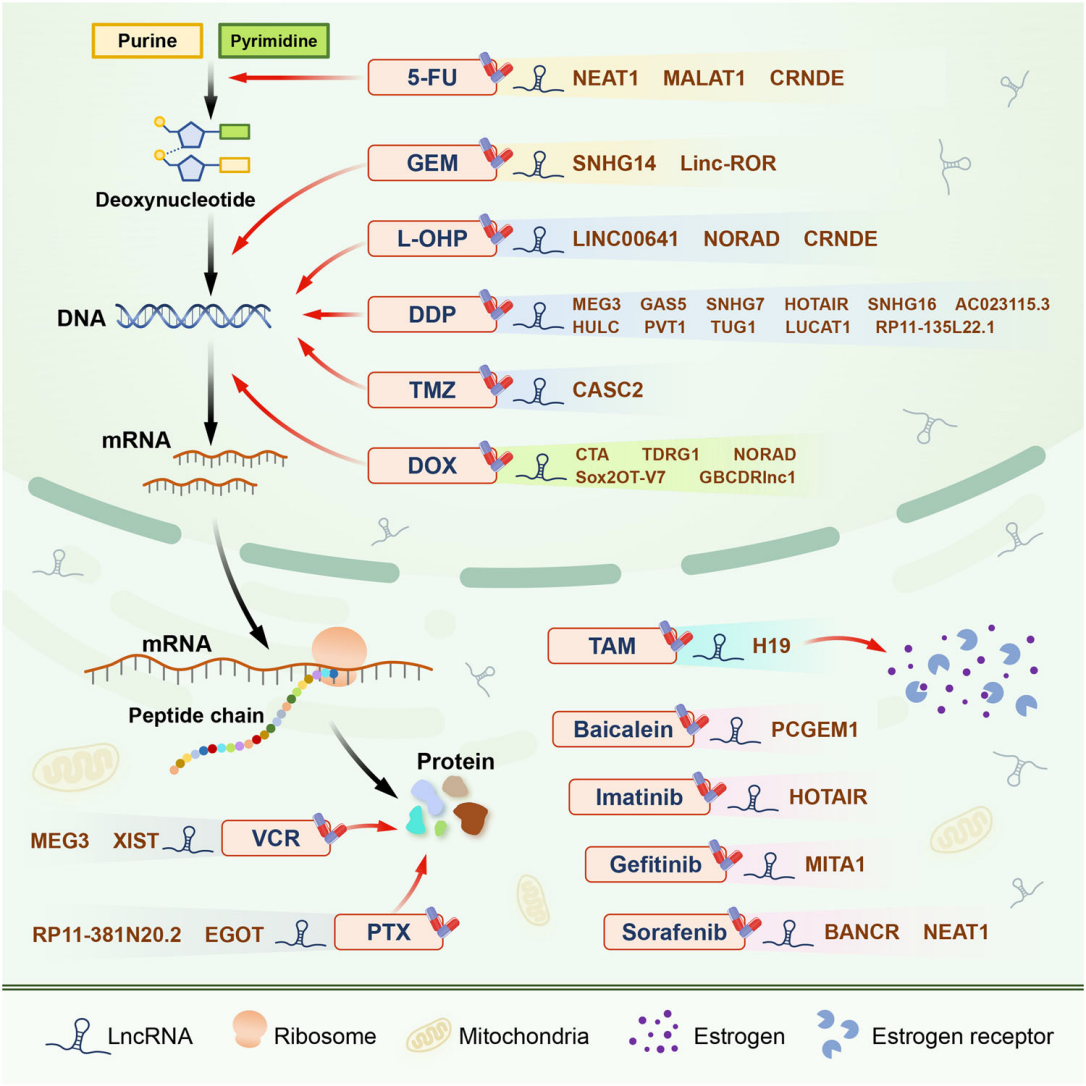
Classification of anti‐cancer drugs and lncRNAs affecting their drug resistance through autophagy. Anti‐cancer drugs can be classified based on their mechanisms of action. These drugs can affect all aspects of gene expression including DNA structure and function, nucleic acid biosynthesis, transcription and RNA synthesis and protein synthesis and function. Additionally, molecularly targeted drugs and hormone‐regulating drugs can also be used to treat certain tumours. LncRNAs have been shown to regulate autophagy in tumor cells and play a role in the development of drug resistance.

There are many different types of anti‐tumour drugs, including cytotoxic drugs that can affect DNA structure and function, interfere with nucleic acid biosynthesis, transcriptional processes, RNA synthesis and protein synthesis and function. There are also non‐cytotoxic anti‐tumour drugs, such as Tamoxifen (TAM), for ER‐positive breast cancer and certain molecularly targeted drugs. Traditional anti‐cancer drugs lack selectivity and can cause significant cytotoxicity and side effects in both cancer cells and normal cells. In recent years, the emphasis of research has shifted towards the development of small‐molecule targeted drugs designed to selectively target cancer cells while preserving the integrity of healthy cells. These targeted drugs offer high efficacy and low toxicity, providing advantages in terms of effectiveness and safety. Furthermore, they contribute to personalized and targeted cancer chemotherapy regimens. However, the development of chemotherapy resistance remains a major challenge that needs to be addressed for all existing drugs (Figure 3).^59^

### 3.1 Drugs that affect DNA structure and function

#### 3.1.1 Cisplatin (DDP)

**FIGURE 4:**
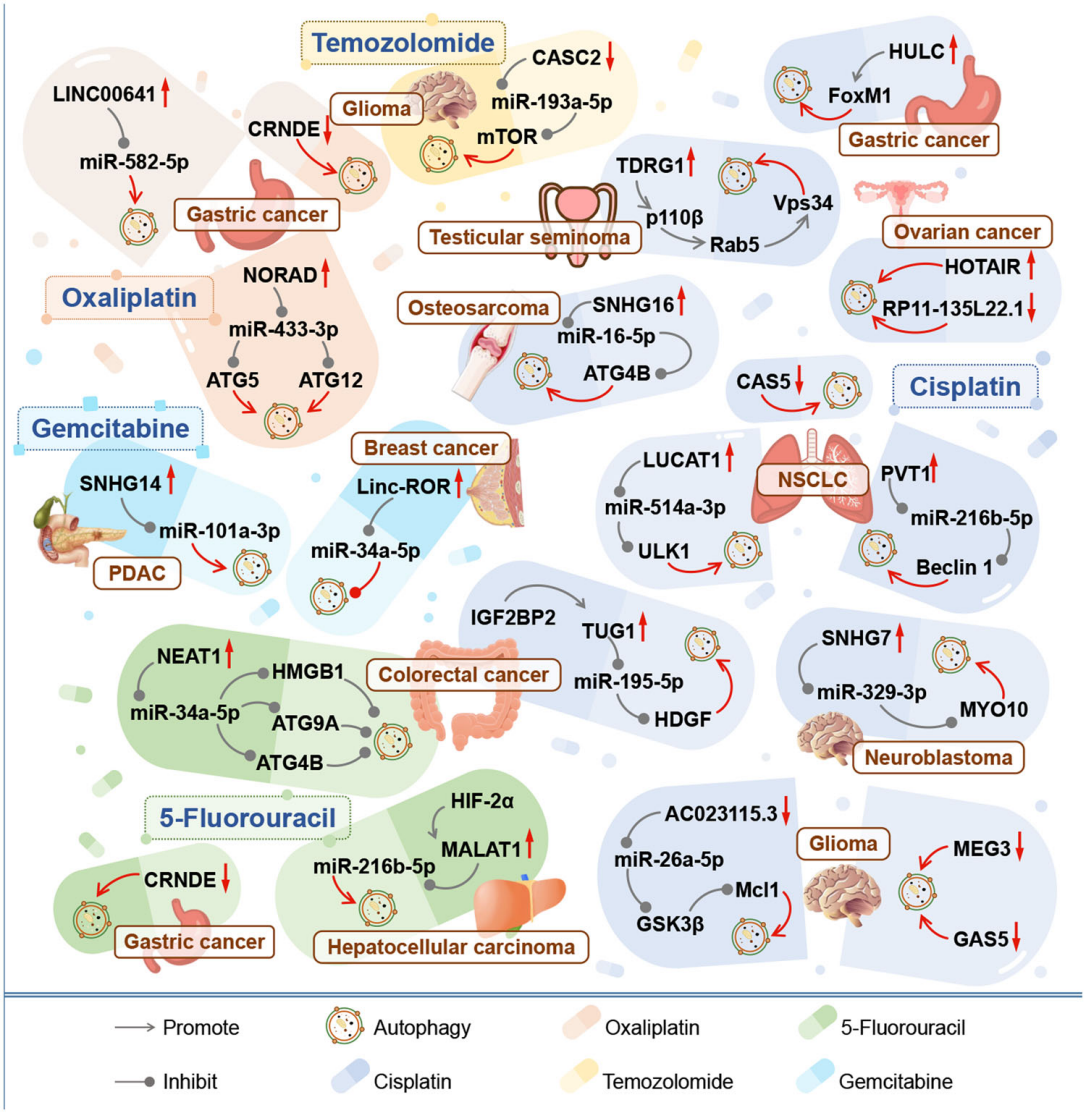
The role of lncRNAs in regulating autophagy and drug resistance to cisplatin, oxaliplatin, temozolomide, 5‐fluorouracil and gemcitabine in tumours. LncRNAs have been shown to regulate autophagy and affect the drug resistance of various tumours to drugs such as cisplatin, oxaliplatin, temozolomide, 5‐fluorouracil and gemcitabine. This includes tumours, such as glioma, neuroblastoma, non‐small cell lung cancer (NSCLC), osteosarcoma, colorectal cancer, testicular seminoma, ovarian cancer, gastric cancer, colorectal cancer, hepatocellular carcinoma, breast cancer and pancreas ductal adenocarcinoma (PDAC).

Cisplatin (DDP) is a first‐generation platinum‐based anti‐cancer drug that is commonly used in chemotherapy for various cancers. It works by irreversibly binding to DNA bases, interfering with DNA replication and transcription and inducing apoptosis‐mediated cell death. However, repeated use of DDP in clinical treatment can lead to the development of chemoresistance in tumour cells.^48^, ^60^, ^61^ In cancers, such as neuroblastoma, glioma, NSCLC, osteosarcoma, colorectal cancer, gastric cancer, testicular seminoma and ovarian cancer, the development of DDP resistance has been linked to an increase in tumour cell autophagy (Figure 4).

In neuroblastoma, the oncogenic lncRNA SNHG7 has been found to act as a sponge for miR‐329‐3p, inhibiting its expression and upregulating MYO10 expression. This promotes neuroblastoma cell autophagy and DDP resistance.^62^ In glioma, Ma et al. discovered that after DDP treatment, lncRNA AC023115.3 was upregulated in the U87 glioma cell line. Elevated AC023115.3 competitively inhibited miR‐26a‐5p, promoting GSK3β expression and Mcl1 degradation in the Bcl2 family. This helped to eliminate DDP‐induced autophagy, enhance apoptosis and increase drug sensitivity in tumour cells.^63^ MEG3 is a tumour suppressor that is downregulated in glioma. DDP treatment induced upregulation of MEG3 expression in U87 cells, reducing autophagy, enhancing apoptosis and increasing drug sensitivity.^48^ GAS5 is another tumour suppressor that was found to be downregulated in four glioma cell lines: U87, U251, U138 and LN18. It was less expressed in U138 and LN18 cells which were less sensitive to DDP.^49^ Huo et al. found that upregulating GAS5 could reduce DDP resistance by activating mTOR signalling and inhibiting excessive autophagy.^49^

In NSCLC, low expression of GAS5 has been linked to higher DDP resistance and poorer clinico‐pathological features. Overexpression of GAS5 in A549/DDP cells can work synergistically with the autophagy inhibitor 3‐MA to inhibit autophagy and restore DDP sensitivity.^64^ Additionally, both lncRNA LUCAT1 and lncRNA PVT1 were found to be highly expressed in NSCLC,^32^, ^33^ particularly in DDP‐resistant cell lines. They upregulate tumour cell viability and autophagy and inhibit apoptosis through the LUCAT1/miR‐514a‐3p/ULK1 axis and PVT1/miR‐216b‐5p/Beclin 1 axis, respectively. This enhances the drug resistance of NSCLC cells to DDP.^32^, ^33^

LncRNA SNHG16 and ATG4B are overexpressed in osteosarcoma tissues and have been linked to lower survival rates.^36^ Liu et al. discovered that SNHG16 can act as a sponge for miR‐16‐5p, upregulating its downstream target gene ATG4B. This promotes autophagy and DDP drug resistance in osteosarcoma.^36^

In colorectal cancer tissues and cells, the expression of lncRNA TUG1 and IGF2BP2 was increased. The RNA‐binding protein IGF2BP2 can bind to and stabilize TUG1, promoting its competitive inhibition of miR‐195‐5p. This leads to the increased expression of downstream genes HDGF and DDX5, amplifying cell proliferation, migration, invasion and autophagy, while suppressing apoptosis and elevating resistance to DDP.^54^ Overexpression of HDGF and DDX5 has also been linked to resistance to the colorectal cancer chemotherapy drugs nordihydroguaiaretic acid and cetuximab, respectively.^65^, ^66^

Methioninase (METase) possesses anti‐tumour characteristics and, when overexpressed, can increase the susceptibility of DDP‐resistant gastric cancer cells to DDP.^67^ Further research has shown that lncRNA HULC can bind to and stabilize the FoxM1 protein in gastric cancer cells, promoting DDP resistance. METase can reduce autophagy in drug‐resistant cells and improve sensitivity by inhibiting the overexpression of HULC and FoxM1.^68^

In testicular seminoma, lncRNA TDRG1 is highly expressed and activates the PI3K/Akt signalling pathway to promote cell proliferation, invasion and autophagy while inhibiting apoptosis and mediating DDP chemotherapy resistance.^69^ PI3K lipid kinases are divided into three classes (I–III), with class III PI3K converting phosphatidylinositol into PI(3)P by combining the catalytic subunit Vps34 with the regulatory subunit Vps15. This is critical for initiating autophagy.^70^ TDRG1 can enhance the interaction between p110β (the catalytic subunit of class I PI3Ks) and the small GTPase Rab5, activating Vps34 by maintaining Rab5 in a GTP‐bound state.^69^

In ovarian cancer tissues, the expression of lncRNA RP11‐135L22.1 was significantly lower than in normal tissues, particularly in the DDP‐resistant group. Low expression of RP11‐135L22.1 has been linked to lower overall survival rates, more advanced tumour stages and larger tumour volumes in ovarian cancer patients. The downregulation of RP11‐135L22.1 was dependent on the time and dose of DDP administration. Combining DDP treatment with overexpression of RP11‐135L22.1 significantly inhibited DDP‐induced autophagy.^60^ The oncogenic lncRNA HOTAIR is upregulated in ovarian cancer. Yu et al. found that combining DDP treatment with knockdown of HOTAIR could also inhibit autophagy and increase DDP sensitivity.^71^

The pervasiveness of DDP resistance in various tumour chemotherapy regimens is evident from the above discussion. These findings robustly establish a connection between the development of DDP resistance and the autophagy process modulated by lncRNAs and their targets. Importantly, this resistance phenomenon is closely linked to poorer prognosis and adverse clinico‐pathological characteristics in specific tumours. Therefore, targeting these specific lncRNAs to interrupt the upstream regulation of the autophagy pathway holds promise as a novel strategy to enhance the effectiveness of chemotherapy.

#### 3.1.2 Oxaliplatin(L‐OHP)

Oxaliplatin (L‐OHP), a third‐generation anti‐cancer medication containing platinum, operates by attaching to and impairing DNA, thereby impeding its replication and delivering its anti‐cancer properties.^61^ Compared to other platinum‐based drugs, L‐OHP is more effective at controlling lesions and has fewer side effects. Using oxaliplatin‐based treatment strategies can improve overall survival rates and reduce local recurrence rates of gastrointestinal tumors.^16^ However, many patients develop chemotherapy resistance after long‐term L‐OHP treatment, which significantly reduces its effectiveness and can lead to relapse and disease progression.^16^

In gastric cancer, both lncRNA LINC00641 and lncRNA NORAD were upregulated, with higher upregulation in L‐OHP drug‐resistant cell lines. This was positively correlated with poor patient prognosis.^16^, ^72^ LINC00641 can promote tumour cell proliferation, migration and autophagy by competitively inhibiting miR‐582‐5p and enhancing L‐OHP drug resistance.^72^ NORAD acts as a sponge for miR‐433‐3p, upregulating the expression of downstream target genes ATG5 and ATG12 to increase autophagic flux and improve tumour cell resistance to L‐OHP.^16^ The expression of lncRNA CRNDE is downregulated in gastric cancer and the degree of downregulation is positively correlated with resistance to chemotherapy drugs.^73^ SRSF6 is an SR family splicing factor that can participate in mRNA alternative splicing.^74^ Zhang et al. found that lncRNA CRNDE can bind to SRSF6 and reduce its stability, reducing the alternative splicing of PICALM mRNA by SRSF6 and decreasing PICALM expression. Downregulated CRNDE in gastric cancer may promote tumour cell autophagy through the SRSF6/PICALM pathway and increase drug resistance (Figure 4).^73^

#### 3.1.3 Temozolomide (TMZ)

TMZ is a small lipophilic molecule and an orally administered imidazolium tetrazine alkylating agent. It can induce 6‐*O*‐methylguanine in DNA, causing it to mismatch with thymine during DNA replication and triggering DNA damage to exert its anti‐tumour effects.^75^ TMZ is extensively employed for the therapy of both initial and recurrent high‐grade gliomas, yet its effectiveness is frequently constrained by the development of drug resistance. Research has shown that TMZ treatment can induce autophagy in glioma cells, promoting chemotherapy resistance.^75^

The tumour suppressor lncRNA CASC2 has been found to be downregulated in glioma tissues and cell lines, which was even more pronounced in patients with advanced clinical stages and those resistant to TMZ.^30^ Downregulation of CASC2 promoted the expression of miR‐193a‐5p and its inhibition of mTOR, significantly increasing the expression of autophagy‐related proteins in cells. This further promoted protective autophagy in tumour cells, resulting in TMZ resistance (Figure 4).^30^

### 3.2 Drugs affecting nucleic acid biosynthesis

#### 3.2.1 5‐Fluorouracil (5‐FU)

5‐Fluorouracil (5‐FU) is an anti‐metabolite drug that is a derivative of uracil with fluorine replacing hydrogen at the C‐5 position. It interferes with essential biosynthetic activities by inhibiting thymidylate synthase (TS) or misincorporating its metabolites into RNA and DNA, inducing cytotoxicity and exerting anti‐tumour effects.^76^, ^77^ As one of the earliest reported chemotherapeutic drugs with anti‐cancer activity, 5‐FU is widely used in chemotherapy for cancers such as colorectal cancer, gastric cancer, liver cancer, breast cancer and oesophageal cancer.^76^ Although 5‐FU‐based chemotherapy has improved patient survival rates to some extent, the emergence of drug resistance has greatly affected its clinical efficacy.^77^ Studies have shown that in gastric cancer,^73^ colorectal cancer^34^ and hepatocellular carcinoma (HCC),^78^ 5‐FU drug resistance is associated with abnormal expression of lncRNAs and increased phagocytosis and autophagy in tumour cells (Figure 4).

In gastric cancer, autophagy induced by lncRNA CRNDE through the SRSF6/PICALM pathway during 5‐FU treatment promoted chemoresistance, similar to L‐OHP.^73^ LncRNA NEAT1 can act as a ceRNA for miR‐34a‐5p and is highly expressed in colorectal cancer tumour tissues and cells. This promotes tumour cell proliferation and protective autophagy, inhibits apoptosis and ultimately enhances resistance to 5‐FU.^34^ MiR‐34a‐3p is a tumour suppressor gene that is underexpressed in many tumors.^79^ Liu et al. found that miR‐34a‐3p can simultaneously target and inhibit multiple targets in colorectal cancer, including HMGB1 and autophagy‐related genes ATG9A and ATG4B, ultimately inhibiting autophagy.^34^ In chemotherapy for HCC, 5‐FU, DOX and mitomycin C (MMC) are commonly used drugs. However, the development of multi‐drug resistance often leads to chemotherapy failure.^78^ Yuan et al. discovered that the oncogenic lncRNA MALAT1 was upregulated in HCC) tissues and cells and was more than twice as highly expressed in the multi‐drug‐resistant BEL‐7402/5‐FU cell line compared to the parental BEL‐7402 cell line.^78^ Further research showed that HIF‐2α can upregulate MALAT1, which can inhibit miR‐216b‐5p, promoting autophagy and increasing tumour cell drug resistance to 5‐FU, DOX and MMC.^78^

#### 3.2.2 Gemcitabine (GEM)

Gemcitabine (GEM) is a synthetic deoxycytidine nucleoside analogue and a prodrug that is activated in tumour cells by deoxycytidine kinase to its active triphosphate form, dFdCTP. dFdCTP competes with dCTP for incorporation into DNA by DNA polymerase during replication, inhibiting DNA synthesis and inducing cell cycle arrest and apoptosis.^80^, ^81^ GEM was first shown to improve survival in patients with pancreas ductal adenocarcinoma (PDAC) in 1997 and remains a cornerstone of chemotherapy for PDAC.^80^ In addition to PDAC, GEM is also an effective chemotherapeutic agent for various solid tumours including breast, ovarian, bladder and NSCLCs. However, resistance to GEM in cancer patients remains a major challenge.

In pancreatic ductal carcinoma, tumour tissues and cells have increased levels of the lncRNA SNHG14. This increase promotes tumour cell growth, migration and invasion while also inhibiting apoptosis and speeding up tumour progression. SNHG14 can also stimulate miR‐101a‐3p and increase the expression of autophagy‐related proteins like RAB5A and ATG4D. This enhances the tumour cells’ resistance to the drug GEM.^82^ In breast cancer, GEM treatment can fight tumours by reducing tumour cell viability and increasing autophagy and apoptosis.^83^ Linc‐ROR exhibits upregulation in human breast cancer MDA‐MB‐231 cells and exerts a negative regulatory effect on miR‐34a‐5p expression. This regulation occurs through the inhibition of histone acetylation in the promoter region of miR‐34a‐5p. Silencing Linc‐ROR leads to the upregulation of autophagy‐related proteins (LC3‐II, Beclin 1 NOTCH1) and the pro‐apoptotic protein p53. Simultaneously, it reduces the expression of the autophagy protein p62 and the anti‐apoptotic protein Bcl‐2, thereby synergistically enhancing the effect of GEM.^83^ Previous research has shown that high levels of Linc‐ROR can cause breast cancer resistance to 5‐FU and PTX by promoting the EMT of tumour cells.^84^ Further research is needed to determine if Linc‐ROR can increase GEM drug resistance by inhibiting autophagy and apoptosis. However, within this pathway, miR‐34a has been identified as a regulator targeting critical autophagy genes including ATG4, ATG5 and ATG9.^34^, ^46^, ^85^ Elevating the levels of miR‐34a offers the potential to effectively target the autophagy process, thereby inhibiting cancer progression. In this pursuit, some researchers have explored the utility of gold nanomaterials, suggesting that the combination of nanomedicine holds promise for achieving this objective (Figure 4).^46^

### 3.3 Drugs that inhibit RNA production by disrupting transcription

#### 3.3.1 Doxorubicin (DOX)

**FIGURE 5:**
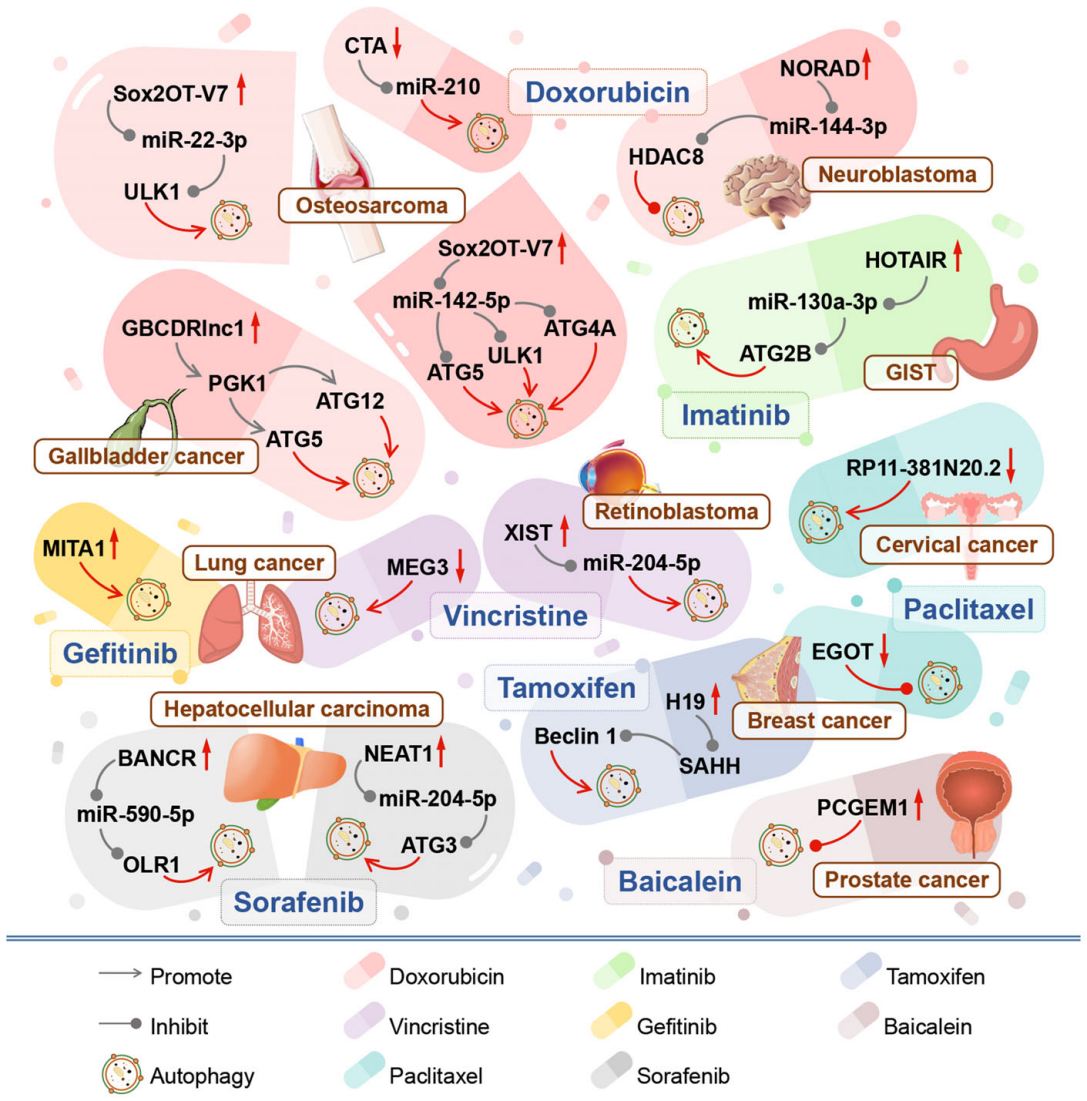
The role of lncRNAs in regulating autophagy and drug resistance to doxorubicin, vincristine, paclitaxel, imatinib, gefitinib, sorafenib, tamoxifen and baicalein in tumours. LncRNAs have been shown to regulate autophagy and affect the drug resistance of various tumours to drugs such as doxorubicin, vincristine, paclitaxel, imatinib, gefitinib, sorafenib, tamoxifen and baicalein. This includes tumours such as osteosarcoma, neuroblastoma, gallbladder cancer, lung cancer, retinoblastoma, cervical cancer, breast cancer, gastrointestinal stromal tumours (GIST), hepatocellular carcinoma and prostate cancer.

DOX, also called adriamycin, is an anthracycline antibiotic from *Streptomyces peucetius* spp.^86^ DOX fights tumours in several ways such as by inserting itself between DNA base pairs, inhibiting DNA polymerase and DNA synthesis. DOX can also inhibit RNA synthesis and transcription by blocking RNA polymerase and can cause single‐ and double‐strand DNA breaks by inhibiting topoisomerase II, limiting DNA replication and transcription.^86^, ^87^ However, DOX resistance is a major issue in tumour treatment. Research has shown that lncRNAs can affect the autophagy process in osteosarcoma,^17^, ^31^, ^88^ gallbladder cancer,^17^, ^38^ and neuroblastoma,^56^ which may be related to DOX resistance (Figure 5).

In osteosarcoma, lncRNA CTA is significantly reduced in tumour tissues and cells and is lower in cell lines exposed to DOX.^88^ Reduced CTA expression correlates with advanced clinical stage and larger tumour size, resulting in shorter survival periods for osteosarcoma patients with low CTA levels compared to those exhibiting high expression.^88^ During chemotherapy, DOX induces autophagy while also activating CTA to increase expression. CTA promotes cell death by binding miR‐210 and inhibiting cell autophagy, restoring some sensitivity to tumour cells.^88^ Wang et al. found that lncRNA Sox2OT‐V7 is increased in osteosarcoma tumour tissues and cell lines, especially in DOX‐resistant tissues. Sox2OT‐V7 can promote autophagy of tumour cells through the Sox2OT‐V7/miR‐22‐3p/ULK1 axis and Sox2OT‐V7/miR‐142‐5p/ULK1, ATG4A and ATG5 axis, mediating drug resistance to DOX.^31^ Epigallocatechin gallate (EGCG), the predominant catechin found in green tea, has demonstrated the ability to alleviate drug resistance in osteosarcoma by targeting Sox2OT‐V7 and inhibiting DOX‐induced pro‐survival autophagy.^17^ Furthermore, recent research has implicated tumour cell autophagy in the maintenance of CSCs, which are believed to contribute to chemotherapy resistance and tumour recurrence.^8^, ^89^ The Notch signalling pathway is recognized for its pivotal role in both tumour development and the maintenance of CSCs and the overexpression of Sox2OT‐V7 further activates this pathway in osteosarcoma stem cells. By targeting Sox2OT‐V7, EGCG partially suppresses the Notch signalling pathway, thus, inhibiting the stemness of osteosarcoma cells and alleviating the DOX‐induced drug resistance to some extent.^17^ However, the direct connection between autophagy and CSCs in the study conducted by Wang et al. lacks direct evidence, highlighting the need for further exploration in future investigations. The lncRNA GBCDRlnc1 is highly expressed in gallbladder carcinoma tissues and is linked to a worse histological grade, more advanced TNM stage and lower overall survival compared to normal adjacent tissues.^37^, ^38^ GBCDRlnc1 interacts with PGK1 protein, inhibits its ubiquitination and degradation, increases the expression of ATG5‐ATG12 conjugate as a downstream target, promotes tumour cell autophagy and induces DOX resistance.^37^, ^38^ NORAD has been shown to play a role in many tumours. Wang et al. also discovered that NORAD is overexpressed in neuroblastoma tissues and cells and is associated with reduced survival rates, advanced INSS stage and lymph node metastasis in affected patients.^56^ NORAD enhances the expression of the oncogenic HDAC8 by acting as a miR‐144‐3p sponge, thereby stimulating tumour cell proliferation, metastasis and resistance to DOX, while concurrently suppressing apoptosis and autophagy.^56^ However, further research is needed to understand the relationship between autophagy and DOX resistance in neuroblastoma.

Moreover, studies have indicated that in the presence of low concentrations of DOX, transcription factor EB (TFEB) exhibits upregulated expression in LoVo and HeLa cells. This upregulation promotes autophagy by enhancing lysosomal biogenesis and inhibiting the mTOR pathway, thereby mediating multi‐drug resistance in tumour cells.^90^, ^91^ Previous research has established that TFEB can be regulated by LncRNA XXYLT1‐AS2 and lncRNA MALAT1, exerting control over the autophagy process in tumour cells.^92^, ^93^

### 3.4 Drugs that inhibit protein synthesis and function

#### 3.4.1 Vincristine (VCR)

Abnormal cell growth is a key characteristic of cancer. Microtubules, a main component of the cytoskeleton, play an important role in cell growth and division.^94^ Vincristine (VCR) is an alkaloid from *Apocycaceae Vinca* that targets microtubules to fight tumours. VCR binds with tubulin to inhibit microtubule polymerization, preventing the formation of spindle filaments and inhibiting cell division.^94^ VCR is used in chemotherapy regimens for various types of malignant tumours due to its effectiveness. However, tumour cells can develop drug resistance over time, reducing its efficacy.^95^

LncRNA MEG3 is expressed at low levels in lung cancer tissues and cells, with significantly lower expression in stage III + stage IV lung cancer patients compared to stage I + stage II.^95^ In lung cancer cell lines A549 and H292, increasing MEG3 expression can reduce the viability and growth of lung cancer cells and enhance the tumour‐inhibiting effects of VCR. MEG3 expression is also significantly lower in VCR‐resistant cells compared to non‐resistant cells, with higher levels of autophagy‐related proteins in drug‐resistant cells.^95^ LnRNA XIST expression is increased in retinoblastoma, with a higher LC3B‐II/I ratio and lower p62 expression in tumour cells, indicating activated autophagy.^62^ Wang et al. found that reducing XIST in retinoblastoma cell lines WERI‐RB1 and Y79 could inhibit autophagy by increasing miR‐204‐5p expression, promoting apoptosis, reducing the half inhibitory concentration value of VCR and increasing tumour cell sensitivity to VCR (Figure 5).^62^

#### 3.4.2 Paclitaxel (PTX)

PTX is a tetracyclic diterpenoid from the bark of the Pacific yew tree. Due to its minimal toxicity, exceptional effectiveness and versatility as a natural anti‐cancer agent, PTX finds extensive application in the treatment of breast cancer, uterine cancer and ovarian cancer.^57^ Unlike VCR, PTX promotes microtubule polymerization and stabilization and inhibits microtubule de‐polymerization. This activates the mitotic checkpoint (also known as the spindle assembly checkpoint) and induces mitotic arrest, producing anti‐tumour effects.^96^

In cervical cancer tissues, lncRNA RP11‐381N20.2 expression is significantly lower than in normal tissues, particularly in the PTX‐resistant group compared to the PTX‐sensitive group.^97^ Low RP11‐381N20.2 expression is also positively linked to poor prognosis in cervical cancer patients, with advanced tumour stage, larger tumour volume and shorter overall survival time. In cervical cancer, PTX can inhibit tumour cell growth and promote apoptosis in a dose‐dependent manner. However, as the use time and dose of PTX increase, tumour cell autophagy is induced and activated and RP11‐381N20.2 expression is reduced, promoting drug resistance. In vitro experiments have shown that increasing RP11‐381N20.2 expression in PTX‐resistant cells can restore some cell sensitivity by inhibiting autophagy.^97^ Eosinophil granule ontogeny transcript (EGOT), an anti‐sense intronic lncRNA expressed by ITPR1, is reduced in breast cancer and positively linked to advanced stage and poor prognosis.^57^ Xu et al. discovered that EGOT can increase ITPR1 expression through both *cis* and *trans* pathways. On one hand, EGOT induces ITPR1 protein expression by forming pre‐ITPR1/EGOT dsRNA. On the other hand, EGOT can also promote pre‐ITPR1 alternative splicing by recruiting the splicing factor hnRNPH.^57^ Specifically, increasing EGOT and ITPR1 expression enhances tumour cell autophagy and apoptosis and increases tumour cell sensitivity to PTX (Figure 5).^57^

### 3.5 Molecularly targeted drugs

#### 3.5.1 Imatinib

Imatinib, a small molecule inhibitor of tyrosine kinase signalling enzymes, was the first kinase inhibitor approved by the FDA.^98^ Imatinib targets the BCR::ABL1 fusion gene and inhibits Abelson tyrosine kinase, making it widely used to treat chronic myeloid leukemia (CML). Imatinib has also been discovered to impede the activity of receptor tyrosine kinases (KIT) and platelet‐derived growth factor receptor alpha (PDGFRA), both of which are frequently associated with activating mutations responsible for gastrointestinal stromal tumours. This makes imatinib effective against these tumors.^98^ Research has shown that imatinib can significantly extend overall survival in patients with gastrointestinal stromal tumours, but drug resistance can develop during treatment.^99^

In gastrointestinal stromal tumours, the expression of the lncRNA HOTAIR is significantly elevated and this expression is further amplified following imatinib treatment.^100^ MiR‐130a‐3p can bind to the complementary sequence of the 3′‐untranslated region (UTR) of autophagy‐related protein ATG2B and inhibit its translation, regulating its protein synthesis post‐transcriptionally. In gastrointestinal stromal tumours, imatinib activates autophagy through the HOTAIR/miR‐130a‐3p/ATG2B axis, inhibits apoptosis and promotes drug resistance (Figure 5).^100^ HOTAIR exhibits high expression levels in CML and is positively correlated with the expression of multi‐drug resistance protein 1. Moreover, imatinib‐resistant cells exhibit significantly higher levels of HOTAIR expression compared to the control group. Silencing HOTAIR has been shown to effectively increase sensitivity to imatinib and enhance the rate of apoptosis.^101^ Additionally, HOTAIR knockdown can reduce the phosphorylation of PI3K/AKT/mTOR proteins, which are implicated in CML drug resistance.^101^, ^102^ Studies have also revealed that HOTAIR promotes the methylation of phosphatase and tensin homolog (PTEN) by interacting with DNA methyltransferases (DNMT1), leading to increased invasion and migration of CML cells while inhibiting apoptosis.^103^ Although direct evidence linking HOTAIR to autophagy in the tumour promoting and drug resistance effects of CML is currently lacking, it is worth exploring the relationship between HOTAIR and autophagy considering the close association between the PI3K/AKT/mTOR pathway and apoptosis with autophagy.^27^, ^104^ Furthermore, in the context of CML, a stem cell‐driven blood cancer, imatinib treatment has been shown to induce autophagy, which promotes the survival of CML stem cells. Conversely, autophagy inhibitors can effectively induce cell death and reduce drug resistance in CML.^105^, ^106^

#### 3.5.2 Gefitinib

Gefitinib, an oral inhibitor of the epidermal growth factor receptor (EGFR) tyrosine kinase, was initially employed in the late 1990s as a form of molecularly targeted therapy for NSCLC.^107^ NSCLC accounts for about 85% of lung cancer cases and is the main subtype of lung cancer.^107^ At diagnosis, nearly 80% of NSCLC patients are already in advanced stages and the 5‐year survival rate is only about 15%, with most having activating EGFR mutations. Although gefitinib is used as a first‐line treatment for NSCLC, acquired drug resistance eventually becomes a problem.^108^

The oncogene lncRNA MIAT1 is highly upregulated in gefitinib‐resistant cell lines compared to parental NSCLC cell lines. Hu et al. discovered that gefitinib treatment can increase LC3II/I and Beclin‐1 expression and decrease p62 expression in NSCLC cells. Overexpressing MITA1 can further promote autophagy and cell viability while inhibiting apoptosis, mediating gefitinib resistance (Figure 5).^108^

#### 3.5.3 Sorafenib

Sorafenib is a multi‐targeted TKI that can inhibit tumour cell growth by blocking the Ras/Raf/MEK/ERK signalling pathway. It can also inhibit tumor angiogenesis by targeting proteins such as platelet‐derived growth factor receptor (PDGFR‐β), vascular endothelial growth factor receptor and hepatic cytokine receptor (c‐KIT).^109^, ^110^ In advanced HCC, sorafenib is used as a first‐line treatment and extends the overall median survival of patients with advanced HCC. However, only about 30% of patients benefit from sorafenib and typically acquire resistance within 6 months.^110^ Research has shown that autophagy activation may be involved in the development of HCC resistance to sorafenib.^109^, ^110^

LncRNA NEAT1, which has been shown to play a carcinogenic role in various cancers, is also highly expressed in HCC and can promote tumor cell growth, metastasis and autophagy. Increased NEAT1 expression is positively linked to poor prognosis in patients.^35^ In the HCC cell lines HepG2 and Huh7, sorafenib treatment inhibits tumor cell growth. Overexpressing NEAT1 competitively inhibits miR‐204‐5p and increases ATG3 expression, promoting autophagy to reduce sorafenib efficacy and induce tumor cell drug resistance.^35^ LncRNA BANCR (also known as LINC00586) is overexpressed in HCC and linked to larger tumor volume, later TNM stage and shorter overall survival.^111^ Compared to the parental cell line, BANCR is significantly overexpressed in the sorafenib‐resistant cell line with increased Beclin‐1 levels and LC3‐II/LC3‐I ratio and decreased p62 levels. Further research showed that BANCR promotes downstream oxidized low‐density lipoprotein receptor 1 (OLR1) expression by targeting and inhibiting miR‐590‐5p, inducing protective autophagy and sorafenib resistance in cells.^112^ Additionally, Zhou et al. found that Rutin, a component from the traditional Chinese medicine Potentilla discolour Bunge, can inhibit autophagy by blocking the BANCR/miR‐590‐5p/OLR1 axis in HCC, restoring some tumor cell sensitivity to sorafenib (Figure 5).^112^

### 3.6 Others

#### 3.6.1 Tamoxifen(TAM)

TAM is a non‐steroidal anti‐oestrogen medication capable of competitively blocking oestrogen binding to the oestrogen receptor (ER) within breast tissue. This blocks ER‐mediated stimulation signals, leading to cell growth arrest.^15^, ^113^, ^114^ TAM represents the pioneering targeted drug for both the treatment and prevention of breast cancer and is extensively employed in clinical practice for managing patients with ER‐positive breast cancer. It can significantly reduce patient recurrence rates and mortality.^115^, ^116^ Research has shown that autophagy is a potential mechanism of TAM drug resistance and overexpressing Beclin 1, a key autophagy player, can promote TAM drug resistance. However, using autophagy inhibitors such as 3‐MA and HCQ to inhibit autophagy‐related genes like ATG5, ATG7 and Beclin 1 can re‐sensitize TAM‐resistant tumor cells.^113^, ^117^

LncRNA H19, an imprinted gene transcribed only from maternally inherited alleles, is highly expressed during embryonic development but repressed shortly after birth.^118^ In various tumours, including breast cancer, H19 has been documented to exhibit anomalous overexpression, facilitating tumor cell proliferation, migration, invasion and the induction of EMT.^119^ Sun et al. observed that oestrogen can elicit H19 expression in a manner dependent on both time and dosage. They also found that H19 expression in the ER‐positive breast cancer cell line (MCF‐7) was notably higher than in the ER‐negative breast cancer cell line (MDA‐MB‐231).^120^ Additionally, Wang et al. showed that in ER‐positive breast cancer cell lines, H19 can promote autophagy and enhance tumor cell drug resistance to TAM by increasing Beclin 1 expression (Figure 5).^15^

#### 3.6.2 Baicalein

Baicalein is a monomer primarily extracted from the root of *Scutellaria baicalensis* that can play a role in treating and preventing cancer including breast cancer, bladder cancer, cervical cancer, HCC, lung cancer, ovarian cancer, osteosarcoma and gallbladder cancer. Research has shown that baicalein can induce tumor cell apoptosis, initiate autophagy, inhibit tumor invasion and metastasis and cause cell cycle arrest.^121^

LncRNA PCGEM1 was initially discovered to be overexpressed in prostate cancer and to promote tumor growth. It has since been found to have a similar effect in other cancers.^122^, ^123^ Han et al. silenced PCGEM1 in LNCaP prostate cells and treated them with baicalein. They found that reducing PCGEM1 expression enhanced the inhibitory effect of baicalein on tumor cell proliferation and colony formation, induced apoptosis and autophagy and increased the sensitivity of LNCaP cells to baicalein.^58^ However, more research is needed to understand the relationship between PCGEM1, baicalein sensitivity or drug resistance and tumor cell autophagy, as well as the specific regulatory mechanism (Figure 5).

## 4 LncRNA CONTRIBUTES TO DRUG RESISTANCE BY REGULATING AUTOPHAGY THROUGH EPIGENETIC CHANGES

**FIGURE 6:**
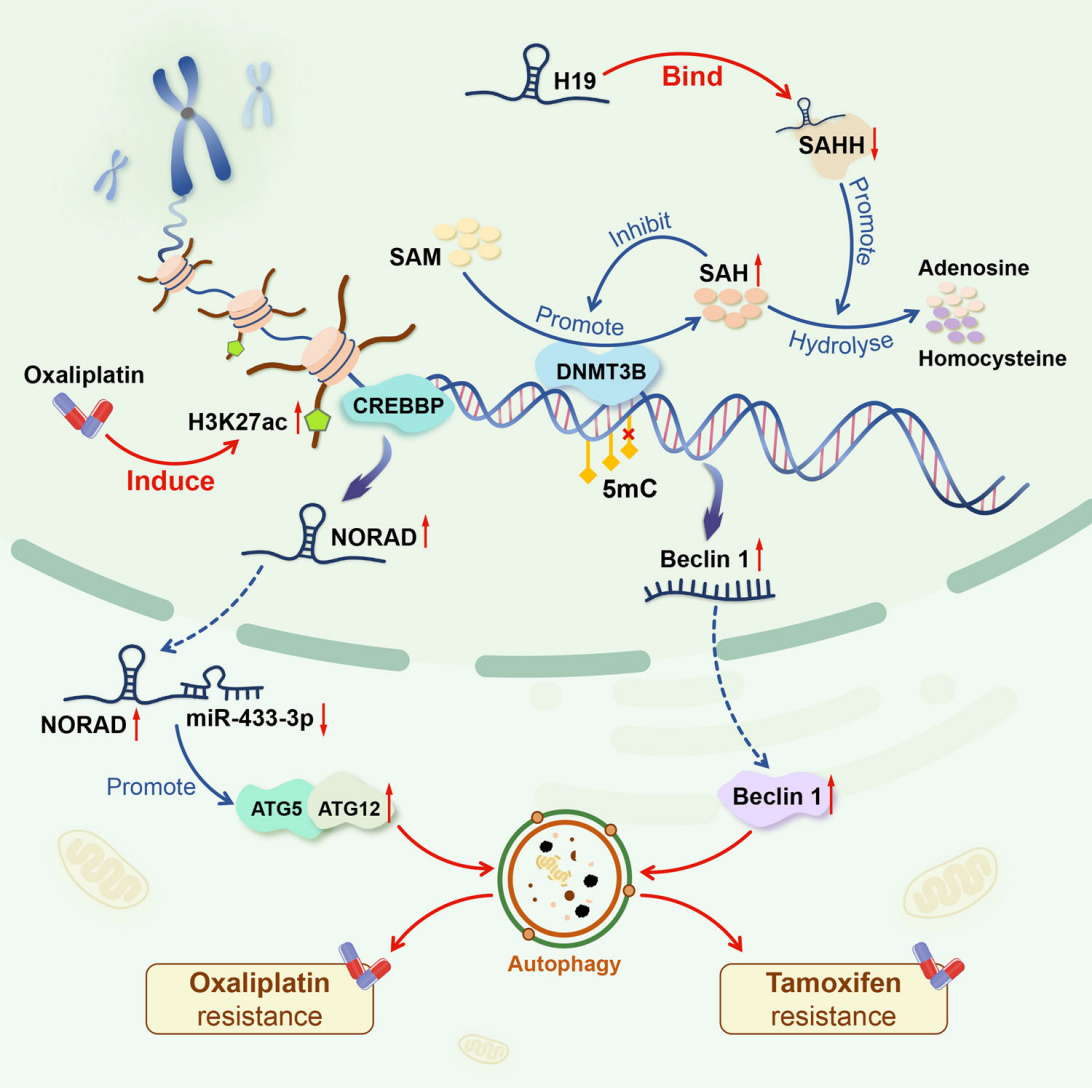
The role of lncRNAs in regulating autophagy and drug resistance through epigenetic modification. In ER‐positive breast cancer, the lncRNA H19 can mediate resistance to tamoxifen by inhibiting DNMT3B‐mediated methylation in the beclin1 promoter region, leading to upregulation of beclin 1 and promotion of tumor cell autophagy. In gastric cancer, H3K27ac enrichment in the promoter region of NORAD leads to upregulation of NORAD expression. In the cytoplasm, NORAD promotes autophagy and resistance to oxaliplatin through the miR‐433‐3p/ATG5, ATG12 axis.

As the field of epigenetics has grown, researchers have discovered that lncRNAs may play a role in drug resistance by affecting autophagy through DNA methylation and histone acetylation (Figure 6).

### 4.1 Tamoxifen (TAM)

In mammals, DNA methylation primarily occurs at the fifth carbon of cytosine to produce 5‐methylcytosine. This is achieved through the combined action of three *S*‐adenosylmethionine (SAM)‐dependent DNA methyltransferases (DNMT1, DNMT3A and DNMT3B). DNMT3A and DNMT3B are responsible for establishing new methylation patterns, while DNMT1 maintains methylation during DNA replication.^124^ During DNA methylation, DNMTs catalyse the transfer of a methyl group from SAM to DNA, producing *S*‐adenosylhomocysteine (SAH) as a byproduct.^124^ SAH is a potent inhibitor of DNMTs and is hydrolysed to homocysteine and adenosine by *S*‐adenosylhomocysteine hydrolase (SAHH) to relieve its inhibitory effect.^125^

Anti‐oestrogen drugs, such as TAM, are commonly used to treat ER‐positive breast cancer patients. However, drug resistance after long‐term use is a major challenge.^15^, ^114^, ^124^ Studies have shown that in ER‐positive breast cancer, the lncRNA H19 can induce autophagy and increase drug resistance by regulating the methylation of specific downstream genes.^15^ H19 is significantly upregulated in ER‐positive breast cancer tissues and cell lines, particularly in TAM‐resistant groups.^15^, ^125^ Wang et al. found that H19 inhibits SAHH by directly binding to it, leading to an accumulation of SAH. This limits DNMT3B‐mediated methylation in the Beclin 1 promoter region and induces upregulation of Beclin 1, promoting tumor cell autophagy and TAM resistance. Knocking down H19 enhances the interaction between DNMT3B and the Beclin 1 promoter region, reducing Beclin 1 expression and TAM‐induced autophagy activity and restoring sensitivity to some extent. This provides a new avenue for overcoming TAM drug resistance.^15^

### 4.2 Oxaliplatin (L‐OHP)

Histones constitute the primary constituents of nucleosomes, wherein an octamer is created by the association of four core histones (H3, H4, H2A and H2B) encircling a DNA segment comprising 147 base pairs.^126^ CREB‐binding protein (CREBBP) and p300 are two acetyltransferases in humans and most‐higher eukaryotes that transfer acetyl groups to lysine residues on histone tails. Due to their high sequence similarity, they are often referred to as p300/CREBBP.^127^ Histone H3 lysine 27 acetylation (H3K27ac) typically occurs at transcription initiation sites and interacts with active enhancers to promote target gene expression, with p300/CREBBP acting as a transcriptional co‐activator.^16^

L‐OHP is a widely used platinum‐based chemotherapy drug for treating advanced gastric cancer. However, the development of drug resistance limits its effectiveness.^128^ Wang et al. found that in L‐OHP‐resistant cell lines, H3K27ac was enriched in the NORAD promoter region and CREBBP expression was higher than in parental cells.^16^ Further experiments showed that continuous exposure to L‐OHP induces DNA damage repair responses in tumor cells, activating CREBBP in the NORAD promoter region and inducing H3K27ac, leading to upregulation of NORAD expression. NORAD can sponge miR‐433‐3p in the cytoplasm, stabilizing ATG5‐ATG12 coupling and enhancing autophagic flux, resulting in L‐OHP resistance.^16^

## 5 THE INTERPLAY BETWEEN AUTOPHAGY AND APOPTOSIS PLAYS A ROLE IN ANTI‐CANCER DRUG RESISTANCE

Apoptosis and autophagy are two key processes that control cell survival or death.^33^ Most cancer therapies work by inducing apoptosis, which kills cells, characterized by pigment aggregation, nuclear fragmentation and caspase activity.^47^, ^129^ There is a complex relationship between apoptosis and autophagy at the molecular level. For example, the BH3 domain within the autophagy‐associated protein Beclin 1 has the capacity to engage with the anti‐apoptotic protein Bcl‐2. Bcl‐2 can form a binding interaction with Beclin 1, leading to the inhibition of autophagy. This inhibition occurs through the prevention of the Beclin 1–class III PI3K complex formation. During drug resistance, induced autophagy can disrupt the Beclin 1/Bcl‐2 complex, releasing Bcl‐2 to enhance cell survival.^47^ Caspases, the main mediators of apoptosis and cell death, can also participate in the inhibition or promotion of autophagy. Autophagy can be inhibited by caspase‐mediated cleavage of Beclin 1, but in some cases, caspases may promote autophagy. The specific mechanism is still being studied.^21^ Additionally, Atg5 is cleaved by stress‐activated cysteine proteases and participates in apoptosis initiation.^130^ Atg12, when unbound, can associate with and render mitochondrial Bcl‐2 family proteins inactive.^131^

A intricate interplay exists between apoptosis and autophagy, potentially contributing to the emergence of resistance to anti‐cancer drugs in cancer development. Studies have shown that autophagy, which is protective in most tumours, can mediate resistance to apoptosis, promoting drug resistance and even leading to refractory cancers.^47^ In tumours where autophagy promotes drug resistance, tumor cell autophagy is mediated by drugs and/or abnormal expression of lncRNAs and is often accompanied by a decrease in apoptosis. For instance, in glioma, the downregulation of CASC2 promotes autophagy through the miR‐193a‐5p/mTOR pathway, resulting in resistance to TMZ. Inhibition of autophagy, overexpression of CASC2, or treatment with miR‐193a‐5p inhibitors enhances apoptosis and increases the sensitivity of glioma cells to TMZ.^30^ In retinoblastoma, XIST is highly expressed, and silencing XIST attenuates cell proliferation and autophagy, thereby enhancing the sensitivity to VCR. Moreover, XIST silencing inhibits the expression of Bcl‐2, promotes the expression of Bax and enhances the activities of caspase‐3 and caspase‐9.^62^ Another study demonstrated that in glioblastoma cells with wild‐type p53, the combination of autophagy inhibitors, such as CQ and TMZ, induces cell death through apoptosis. In contrast, in cells with mutant p53, cell growth is inhibited by G2/M arrest.^75^

However, it should be noted that not all instances of autophagy inhibition in the context of lncRNA and anti‐cancer drug resistance lead to enhanced apoptosis. For example, in TAM‐resistant breast cancer, increased autophagy was not associated with a significant alteration in apoptosis levels.^15^ Conversely, in GEM‐resistant breast cancer, the silencing of highly expressed linc‐ROR in tumor cells can suppress cell viability while simultaneously inducing both autophagy and apoptosis, ultimately leading to cell death and overcoming drug resistance.^83^ Similarly, in DOX‐resistant neuroblastoma with upregulated NORAD and PTX‐resistant breast cancer with downregulated EGOT,^56^, ^57^ both autophagy and apoptosis levels were lower than in sensitive groups.

Overall, the role of apoptosis and autophagy in the development of resistance to different tumours and drugs, as well as their interaction mechanisms, is complex and requires further study. When developing drugs targeting autophagy, it is crucial for researchers to consider their impact on tumor cell apoptosis. The effects on both autophagy and apoptosis should be taken into account to fully harness their positive anti‐tumor potential. This highlights the importance of considering other biological behaviours of tumor cells, beyond autophagy alone, in the research and design of autophagy‐targeted drugs. For instance, inhibiting later stages of autophagy, such as lysosomal function, may concurrently suppress tumor cell macropinocytosis, which has been previously implicated in promoting tumor metabolism and growth.^5^, ^132^

## 6 CONCLUSIONS AND PERSPECTIVES

Mounting evidence suggests that autophagy has a dual impact, serving both as a contributor to tumor progression and as a factor in the emergence of resistance to anti‐cancer drugs.^6^, ^41^, ^42^, ^47^ The role of autophagy is influenced by factors, such as tumor type and stage, the tumor microenvironment and different stress conditions.^6^, ^41^, ^42^ However, the specific conditions and mechanisms of action are not yet fully understood. Given the importance of autophagy, developing anti‐tumor drugs that target this process has become a major research focus and has yielded some results. Although there are a few drugs that target the autophagy process, clinically used drugs, such as CQ and HCQ, primarily concentrate on inhibiting the fusion between autophagosomes and lysosomes during the later stages of autophagy.^1^, ^48^ Drugs targeting other steps in the autophagy process are still under investigation. Furthermore, there remains a scarcity of studies focusing on the therapeutic potential of promoting autophagy. As research in this area continues to advance, there is an urgent need to expand investigations in this field. Our study demonstrates that precise modulation of autophagy through the targeting of lncRNAs holds great promise as a therapeutic approach. In this era of on‐going advancements, novel strategies for early tumor screening and diagnosis are being explored through extensive research. We also aspire to utilize lncRNAs as biomarkers and targets for autophagy modulation, enabling the early detection and treatment of tumours while preventing initial tumor progression or metastasis.

The development of drug resistance in anti‐cancer treatment poses a significant clinical challenge, significantly impacting drug efficacy. This challenge is especially pressing in addressing drug resistance issues associated with commonly used chemotherapy agents like DDP, DOX, 5‐FU and L‐OHP across diverse cancer types. As lncRNAs have garnered attention for their roles in tumours, a substantial body of evidence supports their involvement in regulating autophagy and drug resistance.^53^, ^54^ Furthermore, certain lncRNAs have been concurrently implicated in chemotherapy regimens involving multiple distinct drugs, exemplified by CRNDE in L‐OHP and 5‐FU,^73^ as well as NORAD in L‐OHP and DOX.^16^, ^56^ This discovery opens up a new avenue of research, enabling the identification of specific lncRNAs that regulate tumor cell autophagy and drug resistance. These lncRNAs can potentially serve as markers for this type of tumor. When formulating chemotherapy regimens, it becomes essential to consider inhibiting or increasing the expression of these identified lncRNAs. Furthermore, the dual role of autophagy in tumours highlights the importance of timing and precision in targeting lncRNA‐related autophagy‐targeting drugs. Building upon thorough research, it may be possible to target multiple lncRNAs at different stages of tumor development to precisely modulate the autophagy level of tumor cells. This approach can maximize therapeutic efficacy while reducing or preventing resistance to combined drug therapies. The integration of chemotherapy drugs and targeted lncRNAs has the potential to provide more durable and personalized chemotherapy effects, ultimately improving patient prognosis and increasing survival rates. We summarize current research on anti‐cancer drug resistance targeting lncRNAs and autophagy and discuss their complex regulatory relationships (Table 1). Furthermore, considering that circRNAs form closed‐ring structures through covalent bonds and can function as sponges for miRNAs, a complex mutual regulatory interplay unfolds among lncRNAs, circRNAs and miRNAs. These interactions may give rise to synergistic or antagonistic effects on tumor autophagy. Consequently, we anticipate further research in this domain to illuminate the nuances of this regulatory network and contribute to its refinement in the future. We also found that autophagy and apoptosis have a close but complex relationship during drug resistance, but this relationship requires further study. The design of autophagy‐targeting drugs should consider their impact on other biological behaviours, ensuring a comprehensive evaluation of their therapeutic effects on patients. Moreover, autophagy has been identified as closely associated with drug resistance mediated by CSCs, thus, suggesting a potential new research direction for targeting autophagy through lncRNA modulation.^8^ However, the detection of autophagy levels necessitates the development of more effective and appropriate pharmacodynamic biomarkers that can accurately depict the dynamic changes in autophagy. We hope our research will contribute to the optimization of tumor treatment strategies and improve treatment outcomes for cancer patients.

**TABLE 1:** Anti‐cancer drug resistance is closely related to the effect of lncRNAs on autophagy.

Drug	Tumor type	Clinical cases	Experimental design	LncRNA	Expression (in tumor)	Upstream and downstream genes	Effect of lncRNA on autophagy	Autophagy in drug‐resistant cells	Ref.
Cisplatin (DDP)	Glioma		Glioma (U87MG and U251MG)	AC023115.3	Not available	miR‐26a‐5p/GSK3β/Mcl1	Inhibit	↑	^63^
	Glioma		Glioma (U87)	MEG3	↓		Inhibit	↑	^48^
	Glioma		Glioma (U87, U251, U138 and LN18); Normal human astrocyte	GAS5	↓		Inhibit	↑	^49^
	Neuroblastoma	26 pairs of tissues	Neuroblastoma (SK‐N‐AS); Normal (HUVEC)	SNHG7	↑	miR‐329‐3p/MYO10	Promote	↑	^62^
	NSCLC	15 pairs of tissues	NSCLC (H1650, H1299, H1975, A549 and A549/DDP); Normal (HBE)	GAS5	↓		Inhibit	↑	^64^
	NSCLC	30 DDP‐resistant NSCLC tissues, 30 DDP‐sensitive NSCLC tissues and 30 normal tissues	NSCLC (A549); Normal (IMR90)	LUCAT1	↑	miR‐514a‐3p/ULK1	Promote	↑	^32^
	NSCLC	40 pairs of tissues	NSCLC (A549 and A549/DDP); Normal (293T and 16HBE)	PVT1	↑	miR‐216b‐5p/Beclin 1	Promote	↑	^33^
	Osteosarcoma	30 pairs of tissues	Osteosarcoma (SAOS2 and U2OS); Normal (OB3 and HEK293T)	SNHG16	↑	miR‐16‐5p/ATG4B	Promote	↑	^70^
	Colorectal cancer	43 pairs of tissues	Colorectal cancer (LoVo, LS513, HT29, HCT15 and DLD‐1); Normal (FHC)	TUG1	↑	IGF2BP2/TUG1/miR‐195‐5p/HDGF	Promote	↑	^54^
	Testicular seminoma	10 pairs of tissues	Testicular seminoma (TCam‐2); 24 athymic BALB/c nude mice (4‐5 weeks old, male)	TDRG1	↑		Promote	↑	^69^
	Ovarian cancer	64 ovarian cancer tissues and 30 normal tissues	Ovarian cancer (HO8910)	RP11‐135L22.1	↓		Inhibit	↑	^60^
	Ovarian cancer	20 ovarian cancer tissues and 10 normal tissues	Ovarian cancer (SKOV3 and A2780)	HOTAIR	↑		Promote	↑	^71^
	Gastric cancer		Gastric cancer (SGC7901 and MGC‐803); 12 BALB/c nude mice (4‐6 weeks old, female)	HULC	↑	FoxM1	Promote	↑	^68^
Oxaliplatin (L‐OHP)	Gastric cancer	173 pairs of tissues	Gastric cancer (MKN45、SGC7901 and MKN28)	LINC00641	↑	miR‐582‐5p	Promote	↑	^72^
	Gastric cancer	379 pairs of tissues	Gastric cancer (SGC7901、KATO III and GES‐1); 20 nude mice (4 weeks old)	NORAD	↑	miR‐433‐3p/ATG5 and ATG12	Promote	↑	^16^
	Gastric cancer	38 gastric cancer tissues	Gastric cancer (MGC803); 15 NOD/SCID mice (4 weeks old, female)	CRNDE	↓		Inhibit	↑	^73^
Temozolomide (TMZ)	Glioma	32 pairs of tissues	Glioma (U257 and U87)	CASC2	↓	miR‐193a‐5p/mTOR	Inhibit	↑	^30^
5‐Fluorouracil (5‐FU)	Gastric cancer	38 gastric cancer tissues	Gastric cancer (MGC803); 15 NOD/SCID mice (4 weeks old, female)	CRNDE	↓		Inhibit	↑	^73^
	Colorectal cancer	55 pairs of tissues	Colorectal cancer (HT29, HCT8, HCT116, SW480 and SW620); Normal (FHC)	NEAT1	↑	miR‐34a‐5p/HMGB1, ATG9A, ATG4B	Promote	↑	^34^
	HCC		HCC (BEL‐7402)	MALAT1	↑	HIF‐2α/MALAT1/miR‐216b‐5p	Promote	↑	^78^
Gemcitabine (GEM)	Breast cancer		Breast cancer (MDA‐MB‐231); Normal (MCF10A)	Linc‐ROR	↑	miR‐34a‐5p	Promote	Not available	^83^
	PDAC	65 PDAC tissues and 30 normal tissues (from TCGA database)	PDAC (SW1990); Normal (HPDE6C7 and HEK293T)	SNHG14	↑	miR‐101a‐3p	Promote	↑	^82^
Doxorubicin (DOX)	Osteosarcoma	80 ovarian cancer tissues and 12 normal tissues	Osteosarcoma (SAOS2, U2OS and MG63); Normal (human osteoblast)	CTA	↓	miR‐210	Inhibit	↑	^88^
	Osteosarcoma	32 paired of tissues	Osteosarcoma (SAOS2, U2OS, MNNG/HOS Cl #5 and MG63); Normal (hFOB); 23 nude mice	Sox2OT‐V7	↑	miR‐142‐5p/ULK1, ATG4A, ATG5; miR‐22‐3p/ULK1	Promote	↑	^31^
	Neuroblastoma	38 paired of tissues	Neuroblastoma (SK‐N‐SH and IMR‐32); Normal (HUVEC); 8 mice	NORAD	↑	miR‐144‐3p/HDAC8	Inhibit	Not available	^56^
	Gallbladder cancer	45 paired of tissues	Gallbladder cancer (NOZ and GBC‐SD); 10 nude mice (4 weeks old, male)	GBCDRlnc1	↑	GBCDRlnc1/PGK1/ATG5, ATG12	Promote	↑	^71^, ^72^
Vincristine (VCR)	Lung cancer	The number is not shown	Lung cancer (A549 and H292)	MEG3	↓		Inhibit	↑	^95^
	Retinoblastoma	25 retinoblastoma tissues and 6 matched normal retinal tissues	Retinoblastoma (WERI‐RB1 and Y79); Normal (ARPE‐19); 24 BALB/c nude mice (5‐7 weeks old, male)	XIST	↑	miR‐204‐5p	Promote	↑	^133^
Paclitaxel (PTX)	Cervical cancer	60 cervical cancer tissues and 30 normal tissues (from TCGA database)	Cervical cancer (SiHa)	RP11‐381N20.2	↓		Inhibit	↑	^97^
	Breast cancer	258 paired of tissues	Breast cancer (MCF7, T47D, UACC‐812, SK‐BR‐3, MDA‐MB‐453, MDA‐MB‐231, Hs578T, HCC70, BT549 and MDA‐MB‐468)	EGOT	↓		Promote	↓	^57^
Imatinib	GIST		GIST (GIST‐882 and GIST‐T1); 32 nude mice (about 4 weeks old, male)	HOTAIR	↑	miR‐130a‐3p/ATG2B	Promote	↑	^100^
Gefitinib	NSCLC		NSCLC (HCC827)	MITA1	↑		Promote	↑	^108^
Sorafenib	HCC	68 paired of tissues	HCC (Hep3B, HepG2, Huh7, HCCLM3 and SK‐HEP1); 24 BALB/c nude mice (3‐4 weeks old, male)	LINC00586 (“BANCR”)	↑	miR‐590‐5p/OLR1	Promote	↑	^112^
	HCC		HCC (HepG2 and Huh7)	NEAT1	↑	miR‐204‐5p/ATG3	Promote	↑	^35^
Tamoxifen (TAM)	Breast cancer	23 TAM‐resistant and 14 TAM‐sensitive breast cancer tissues	Breast cancer (MCF7); 20 nude mice (5 weeks old)	H19	↑	H19/SAHH/DNMT3B	Promote	↑	^15^
Baicalein	Prostate cancer		Prostate cancer (LNCaP)	PCGEM1	↑		Inhibit	↓	^58^

GIST, gastrointestinal stromal tumor; HCC, hepatocellular carcinoma; NSCLC, non‐small cell lung cancer; PDAC, pancreas ductal adenocarcinoma; ↑, upregulated; ↓, downregulated.

## AUTHOR CONTRIBUTIONS

CZ, ZX and SD contributed to the conception, design and final approval of the submitted version. CZ and ZX collected and analysed literature. CZ, ZX and SD contributed to manuscript writing. All the authors conceived and gave the approval of the final manuscript.

## FUNDING INFORMATION

The research was supported by Qiantang Scholars Fund in Hangzhou City University.

## CONFLICT OF INTEREST

All authors declare no conflict of interest.

## DECLARATIONS

The authors declare that the study was conducted in the absence of any business or financial relationship that could be interpreted as a potential conflict of interest.

## AVAILABILITY OF DATA AND MATERIAL

Data sharing is not applicable to this article as no new data were created or analyzed in this study.
